# The *Allium cepa* Model: A Review of Its Application as a Cytogenetic Tool for Evaluating the Biosafety Potential of Plant Extracts

**DOI:** 10.3390/mps8040088

**Published:** 2025-08-02

**Authors:** Daniela Nicuță, Luminița Grosu, Oana-Irina Patriciu, Roxana-Elena Voicu, Irina-Claudia Alexa

**Affiliations:** 1 Department of Biology, Faculty of Sciences, “Vasile Alecsandri” University of Bacău, 157, Calea Mărășești, 600115 Bacău, Romania; daniela.nicuta@ub.ro (D.N.); roxana.voicu@ub.ro (R.-E.V.); 2 Department of Chemical and Food Engineering, Faculty of Engineering, “Vasile Alecsandri” University of Bacău, 157, Calea Mărășești, 600115 Bacău, Romania; lumig@ub.ro (L.G.); oana.patriciu@ub.ro (O.-I.P.)

**Keywords:** plant extracts, in vivo test, *Allium cepa* L., toxicity potential

## Abstract

In establishing the safety or tolerability profile of bioactive plant extracts, it is important to perform toxicity studies using appropriate, accessible, and sustainable methods. The *Allium cepa* model is well known and frequently used for accurate environmental risk assessments, as well as for evaluating the toxic potential of the bioactive compounds of plant extracts. The present review focuses on this in vivo cytogenetic model, highlighting its widespread utilization and advantages as a first assessment in monitoring the genotoxicity and cytotoxicity of herbal extracts, avoiding the use of animals for testing. This plant-based assay allows for the detection of the possible cytotoxic and genotoxic effects induced on onion meristematic cells. The outcomes of the *Allium cepa* assay are comparable to other tests on various organisms, making it a reliable screening test due to its simplicity in terms of implementation, as well as its high sensitivity and reproducibility.

## 1. Introduction

In recent years, research on plant extracts has continuously increased, highlighting their importance and use in various fields, including the food, pharmaceutical, and cosmetic industries [1]. An inexhaustibly rich source of bioactive phytocompounds, plant extracts may have multiple therapeutic and pharmacological benefits for human health [2,3].

The biomolecules extracted from plants have excellent potential for replacing synthetic drugs. Plant-based extracts are perceived as an alternative or as a complement to classical medicine, their integration in various treatments being a sustainable strategy [3,4,5,6,7,8].

However, in addition to the extraction, analysis, or isolation of certain bioactive compounds, researchers have been concerned with the toxicity of herbal extracts. As they are a complex mixture of compounds, and due to the possible interaction between certain compounds, the plant extracts are not completely without side effects or toxicity [9]. Even though some extracts are considered beneficial, if consumed in large quantities, they can be toxic. The toxicity of herbal extracts depends on several factors, including the extraction method, chemical composition, dosage, and interactions with other substances.

Therefore, researchers consider it imperative and of great importance to evaluate the cytotoxic potential of plant extracts, to ensure that their use is harmless. Various in vivo and in vitro assay are used for establishing the safety or tolerability profiles of bioactive plant extracts; toxicological methods frequently involve brine shrimp, cell lines or animals [10,11]. Before performing very elaborate, long-term and expensive tests on cell lines and knowing that toxicity assessment involving animal experiments is limited by ethical and economic reasons, more accessible and sustainable assays can be carried out as an initial rapid and low-cost approach in the evaluation of the toxicity.

Higher plants are representative systems that are known to be sensitive indicators to the action of toxic agents. Materials of plant origin, such as whole plants, seeds, organs and tissues, have proven to be suitable for monitoring cytotoxicity when exposed to different chemical agents [12,13].

Plant-based monitoring systems rely on microscopic observations of aberrations occurring during mitosis and the subsequent effects on chromosomes. Therefore, plants such as *Allium cepa*, *Allium sativum*, *Lactuca sativa*, *Sinapis alba*, *Triticum aestivum*, *Vicia faba*, *Zea mays*, etc., possess excellent characteristics for monitoring and screening the cytotoxicity of different agents [12,13,14,15,16,17,18]. The advantages of the plant-based toxicity system are numerous: the ease of obtaining the material, its storage and handling, simplicity and rapidity of implementation, high sensitivity and reproducibility [19,20,21,22,23].

In search of such a method for evaluating the potential toxicity of some plant extracts, following a careful and meticulous bibliographic study, our research team turned its attention to the *Allium cepa* model [24,25].

Due to our successful application of this plant-based system, the present article constitutes an exploratory review of the *Allium cepa* model: in particular, its use as a cytogenetic tool for assessing the biosafety potential of plant extracts. We highlight the advantages it offers.

Moreover, this review aims to highlight the differences between the experimental protocols and the influences of several factors related to both the extraction process for the tested samples and the experimental conditions of the *Allium* test itself on the level of cytogenotoxicity of plant extracts. These aspects are less frequently discussed in the very few reviews already published [21,23,26]. Additionally, this systematic review intends to provide an overview of the plant species studied using this model.

## 2. The *Allium cepa* Model: General Considerations

The *Allium cepa* test is an in vivo experimental model used to evaluate DNA damage (clastogenic and/or aneugenic effects) by identifying chromosomal aberrations and disorders occurring in the mitotic cycle, due to the action of various mutagenic agents.

**Figure 1:**
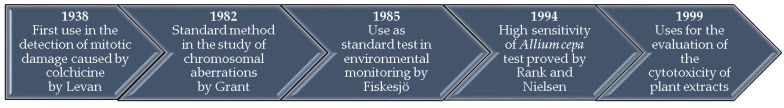
Some important moments in the timeline of the *Allium cepa* bioassay.

Since 1938, when it was used for the first time by Levan [27] when studying the effect of colchicine on the mitosis of onions, the methodology of the *Allium cepa* assay has undergone continuous improvements, making it appropriate for many applications. Important contributions to the development of this bioassay were made by Grant [28,29], Fiskesjö [30,31], Rank and Nielsen [32] (Figure 1). They reported that, when compared to other test materials, *Allium* material can produce similar results. It has also been demonstrated that this model has high sensitivity and can be used as a standard method for environmental monitoring [22,23,33,34].

**Figure 2:**
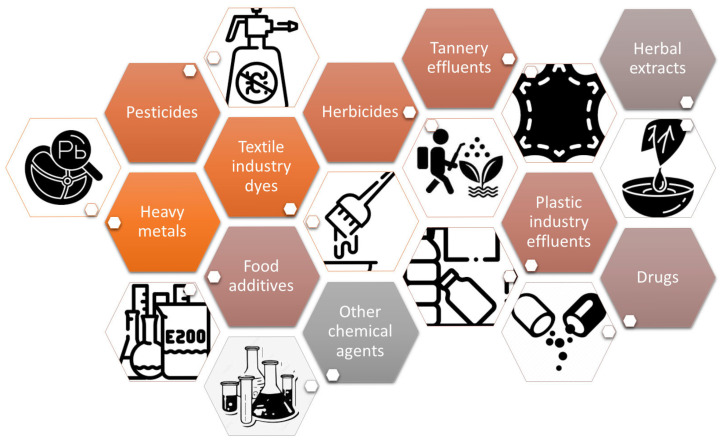
Cytotoxicity screening of different agents using the *Allium cepa* model.

Over the years, the *Allium cepa* model has been used, with good results, in the detection of a wide variety of pollutants and chemical agents (Figure 2).

Several comprehensive reviews concerning the application of the *Allium cepa* as environmental monitoring assay have been reported [21,22,33,34,35]. Research describing the use of *A. cepa* tests in the investigation of heavy metal accumulation in soil, surface water and sediment, industrial wastewater, groundwater, vegetables, etc., are noteworthy [33,34,35,36,37,38].

The *A. cepa* bioassay was successfully applied to effluents from the tannery, textile and plastic industries [39,40,41,42,43]. Several studies are related to the cytotoxicity of some herbicides [44], pesticides [45,46,47,48], fungicides [49], insecticides [50], or other chemical agents [51,52]. Moreover, the potential cytotoxicities of therapeutic drugs (e.g., doxorubicin, erlotibin, metalodrugs, nevirapine, etc.) [53,54,55], food additives (e.g., saccharin, potassium metabisulphite) and animal feeds additives (e.g., urea) [56,57] were tested using the *Allium cepa* assay. Additionally, the *Allium cepa* model was reported “as a ‘warning’ bioindicator in detecting the genotoxicity of medicinal plants” [21,26].

## 3. Basic Principles of the *Allium cepa* Test and Protocol

Toxicity studies based on the *Allium* test are performed on onion roots, which, when exposed to different substances, can indicate their potential cytotoxic or genotoxic effect on organisms.

Onions are considered appropriate for toxicological evaluations because the roots grow rapidly and their tips contain cells in various phases of cell division, showing a clear and rapid response to genotoxic substances; moreover, spontaneous chromosomal damage rarely occurs. Due to the presence of distinct cells, large chromosomes in a reduced number (2*n* = 2*x* = 16), and a stable karyotype, it is easy to identify the possible chromosomal lesions and mitotic cycle disorders under a microscope [21,22,23]. The reduced number of chromosomes (8 pairs) compared with other species (e.g., wheat *Triticum aestivum*: 2*n* = 42) simplifies their tracking and identification during cell division, highlighting possible mutations that can affect the number of chromosomes such as polyploidy (3*x*, 4*x*, etc.) or aneuploidy (2*n* + 1, 2*n* − 1).

**Figure 3:**
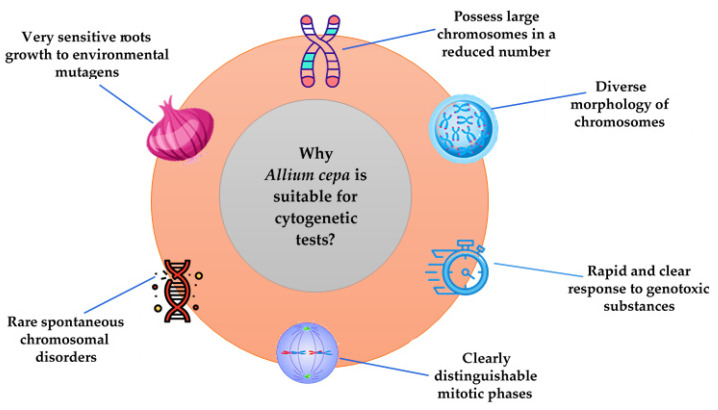
Characteristics of the onion that make it suitable for cytogenetic tests.

Some characteristics of the onion that make it suitable for cytogenetic tests are presented in Figure 3.

Since 1938, when Levan [27] described the first protocol for the *Allium cepa* test, the recommended plant material is represented by the onion bulbs with rapidly growing root tips used in most reported studies. The cells of the root tip actively divide, being the first to come into contact with substances in the environment. Therefore, toxic effects on mitosis and chromosome behavior during the division phases can be observed [58]. Buds from germinated seeds can also be used for the same purpose, as mentioned in other research [59,60]; alternatively, seeds are first germinated in water until the roots are about 2 mm in height [61,62].

The onion bulbs used for testing should be of similar size (approximately 1.5–2.0 cm in diameter) and not exposed to herbicide or fungicide treatments [19,63]. Generally, between three and five onion bulbs are needed for each sample (including the control) to obtain roots. According to the protocol proposed by Tedesco and Laughinghouse [21] as a standard experiment for the *Allium* test, it is recommended to use five different sets of bulbs: one for the negative control, one for the positive control with a known genotoxic agent, and three groups for different concentrations of the test agent. Some authors initially use a larger number of bulbs to test their germination rate, placing them in water for two or four days. Subsets of three or five bulbs which showed the best root growth are then chosen to be exposed to the test solutions [54,64,65,66,67]. It is recommended that the bulbs be lightly scraped in the lower area (primary root ring), to favor the emergence of new roots [19,68]. Many studies report that onion bulbs are initially placed in distilled or tap water (if potable) in narrow glass or plastic containers (50 mL) [21]. Only the area where the roots will form is submerged in water. The water should be renewed every day until the roots grow to a certain length. Root growth varies in time (two to four days), depending on the temperature conditions in which the onion bulbs are stored (a room or growth room/chamber at 22 ± 2 °C) [35]. If onion bulbs are placed in a growth chamber, a controlled photoperiod (18 h/6 h light/dark) can also be ensured. When the roots reach the appropriate length (0.5–2 cm), the onion bulbs may be transferred to the flasks containing the different test extracts and only the base of each bulb should be immersed/suspended in the extract. The exposure time of onion roots in the tested plant extracts may vary: 24 h [69,70], 48 h [65,71], 72 h [68], 96 h [67]. Sabini et al. [72] reported two and five days, as well as two days followed by three days with water (reversion) of exposure.

**Figure 4:**
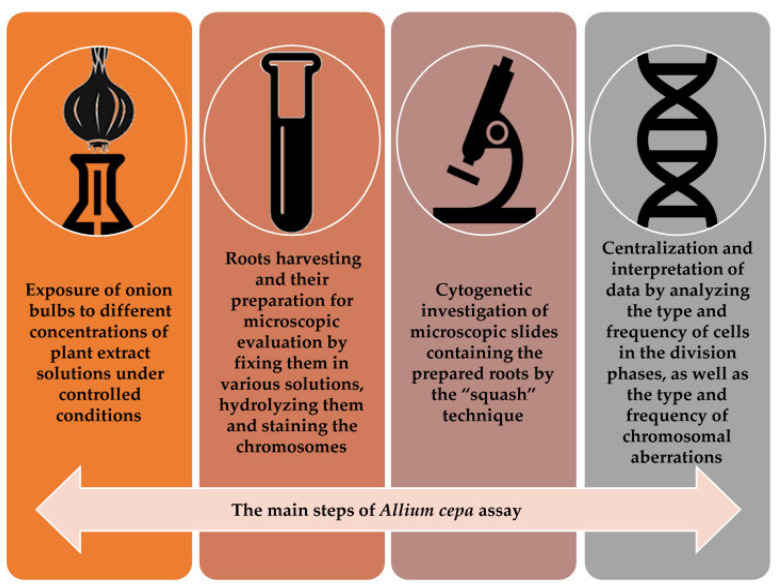
Basic protocol steps for the *Allium cepa* model.

The main steps for the *Allium cepa* protocol are presented in Figure 4.

In the standard *Allium* testing protocol, normal tap water [30,73,74,75], or distilled water [76] are used as the negative control sample. It seems that, compared to tap water, distilled water used as a negative control leads to a statistically significant inhibition of mitosis in the apical cell of the onion [75]. Several studies report the use of a positive control sample, in addition to a negative control sample. Various substances are used as positive controls, namely: glyphosate [70,77,78,79,80,81], cyclophosphamide [71], methotrexate [82,83], paracetamol [72,84], methyl methane sulfonate [85], ethyl methane sulfonate [69,73,74], copper sulfate [86,87], lead nitrate [88], hydrogen peroxide [89], sodium azide [90], dimethyl sulfoxide [91]. Glyphosate, most commonly used as a positive control, is a chemical compound known for its extremely cytogenotoxic effect; it is used as herbicide for a broad spectrum of weeds [92,93]. When the essential oil samples are tested, ethanol or methanol is used as positive control [94,95].

At the end of the exposure period, onion roots treated with the test solutions, including the control, are harvested for cytogenetic preparations. Each plant has its own biological clock for mitotic division; therefore, the root harvesting should be performed when the meristematic cells are actively dividing [96]. The timing of root cutting is therefore a crucial step to identify as many cells as possible in the different phases of mitotic division. The duration of mitosis in roots of *Allium cepa* L. is about 4 h (prophase—2 h, metaphase—40 min, anaphase + telophase − 1 hrs and 20 min) [97]. According to Sangur et al. [96], a high frequency of metaphase cells coincides with a high value of the mitotic index. Therefore, it is advisable to harvest the roots at the time of metaphase unfolding within the mitotic division. Well-growing roots with an average length of 1–2 cm are the best candidates for cytogenetic studies, while exceptionally long or short roots are removed [21,35].

After harvesting, the roots should be immersed in a fixative solution, which has the role of instantly coagulating the cellular constituents. In this way, the cells are rapidly fixed in the phase of mitotic division at that time. A mixture of alcohol and acetic acid can be used for this step, namely, Clarke’s fixative (ethanol:glacial acetic acid, 3:1 *v*/*v*) for 90 min [60,76], Farmer solution (ethanol:glacial acetic acid, 3:1 *v*/*v*) [24,25,98], Carnoy’s solution (ethanol:glacial acetic acid, 3:1 *v*/*v*) [99], or a solution of acetic acid:methanol, 1:3 *v*/*v* [19]. Fixation is achieved by keeping the harvested roots in fixative solutions for 12–18 h at 4 °C in the refrigerator. Until microscopic preparations are made, the roots can be preserved in 70% ethanol in the refrigerator for several months [70,100]. In the protocol described by Wierzbicka [63], fixation can be performed in a solution containing hydrochloric acid (glacial acetic acid (45%):HCl (1N), 9:1 *v*/*v*) by immersing the roots for 5 min at 50 °C. Therefore, together with fixation, hydrolysis of onion roots also occurs [28].

The hydrolysis step is performed with the aim of dissolving the pectocelluloses from the cell wall, allowing the dye used for coloration to penetrate the cell and the chromosomes in the nucleus. In this regard, root hydrolysis can be performed at room temperature with 1N HCl for 20–30 min, or at 60 °C (in a water bath) for 5–10 min. Thus, the root tissue softens, and the dye is able to penetrate the cells, reaching the nucleus to stain the genetic material. Then, the roots are gently rinsed with distilled water, avoiding damage to the hydrolyzed tips. This step is very important because, if HCl is not removed by washing the roots, staining is compromised and the cells cannot be identified under the microscope [68,80,101].

Root coloring is accomplished by treating the roots with different dyes, allowing the microscopic observation of various cell categories in interphase or division. Thus, aceto-orcein solution (2%) [66,67,69,94,101], acetocarmine (2%) [64,68,89] or lacto-propionic orcein [102] ensure a rapid staining in few hours and a good contrast of the chromosomes, which explains their frequent use in the mentioned studies. The cytogenetic preparations must be visualized immediately, because their discoloration occurs over time, which represents a disadvantage of using these staining solutions. For the accuracy of cytogenetic preparations, a staining step with Schiff reagent [55,103] or carbol fuchsin solution [24] is recommended in cytogenetic research, which allow for a very good visualization of nuclei, chromatin and chromosomes in metaphase or anaphase. The microscopic preparations are persistent, and the roots remain stained for a long time (one to two years) if kept in the refrigerator. However, the need for a carefully controlled acid hydrolysis and the longer root staining time (three to five days in the refrigerator) could constitute disadvantages of using these types of coloring agents. Vicentini et al. [104] reported that the roots were fixed and stained with Feulgen reaction.

After root staining, preparation of microscope slides for cell analysis is carried out via the “squash” technique, consisting first in placing one to two onion roots on a microscope slide, in a drop of acetic acid aqueous solution. By cutting the tips of the roots (approximately 1–2 mm) with a scalpel, only the area containing the meristematic cells where mitosis occurs is preserved for microscopic analysis. Then, a coverslip is placed over the tissue and the plant material is crushed by pressing. The cells are thus arranged in a single plane, without overlapping, so that they can be observed individually under the microscope [35,70]. For each bulb, from the harvested and stained roots, two or three slides are prepared for analysis and cell counting under a microscope [19,80,105]. Yekeen et al. [106] reported that, for the fixation and preservation of the samples, the slides can be immersed in liquid nitrogen, thus allowing their subsequent evaluation. For the same purpose, the edges of the slide can be sealed by applying transparent nail polish, so that the cytogenetic preparation can be analyzed even after several days [28,38,72].

One of the objectives of cytogenetic research is to analyze the number and behavior of chromosomes in dividing cells, under the influence of different types of plant extracts.

The analysis of cytogenetic preparations is performed using the 40× and 100× objectives of an optical microscope, which can be equipped with a camera and connected to a computer [67,68,107].

Each microscopic slide is examined to identify and quantify the different cell types present in the microscopic fields. For a correct evaluation, it is recommended to investigate 1000–5000 cells/microscopic slide/per tested sample, as well as in the control samples [30,38,108]. Few authors reported the analysis of a larger number of cells (over 5000) [59,60,66].

**Figure 5:**
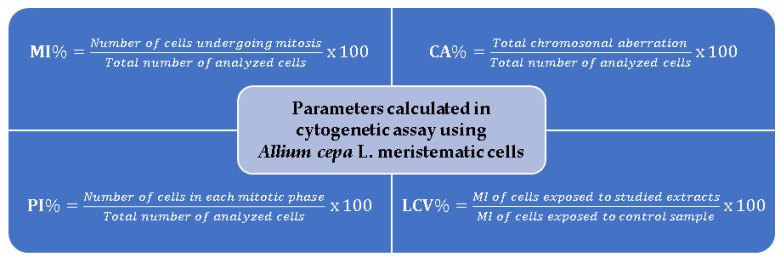
Cytogenotoxic parameters for the *Allium cepa* model.

The most important cytogenetic parameters calculated based on microscopic observations are the mitotic index (MI) and the proportion of chromosomal abnormalities (CA). In some reported studies, in addition to these parameters, the index of each phase of mitosis (PI) is also calculated [25,76,102,109], as well as the limit value of cytotoxicity (LCV) [25,36,40,76]. The calculation formulas of these parameters are detailed in Figure 5.

**Figure 6:**
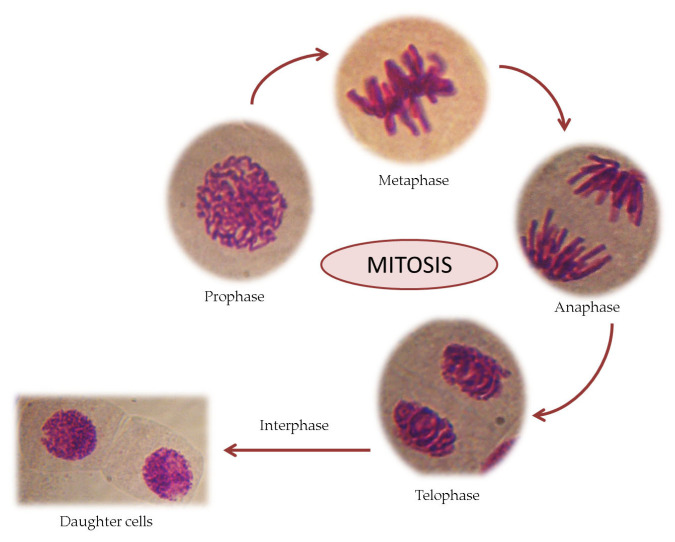
Steps of mitosis in *Allium cepa* L. roots (images from own research).

The mitotic index is a tool used to measure the percentage of dividing cells from each phase of mitosis out of the total cells observed in a microscopic sample. A correct identification of all phases of cell division in *Allium cepa* L. roots is essential. The mitotic cell division comprises regular phases such as prophase, metaphase, anaphase and telophase (see Figure 6). Interphase is a stage in which genetic material is replicated, which takes place over a longer period of the cell cycle compared to mitosis. The most numerous cells are found in the interphase.

Therefore, the number of cells analyzed under the microscope is important for calculating the mitotic index (MI) as accurately as possible. If, in the samples tested with different plant extracts, the MI value is high, this indicates intense cellular activity, and the tissue is actively growing, since it is not affected. If, on the contrary, the MI value is low (compared to the control sample), it indicates the inhibition of this parameter, which can be interpreted as cell death, a delay in the kinetics of cell proliferation, or cellular damage [76], due to the cytotoxic or genotoxic effect of the plant extracts [58,67,68,69,76]. The MI may vary in different roots of the same plant, but the average data are fairly stable [21]. Additionally, the reduced number of cells per division phase (PI) indicates an inhibitory effect on the division process due to tested extracts [65]. Some authors reported that, if the MI value decreases by 50% compared to the control, it is considered the limit value for cytotoxicity (LVC), but, if it drops below 22–25%, it can be lethal to organisms [25,36,40,76].

Chromosomal aberrations (CAs) are changes in the structure and number of chromosomes and can be observed in all stages of mitosis. The most remarkable cells can be highlighted; especially in anaphase and telophase, CAs appear in the form of chromosome bridges, chromosome losses and fragments, chromosome delays, disorganized and multipolar anaphases (star anaphase), and c-mitosis. A series of chromosomal aberrations can also be highlighted in the metaphase as a result of the expulsion of whole chromosomes from the metaphase plate, as well as chromosome breaks, chromosome fragments, and irregular metaphases [110]. Chromosomal aberrations are caused by the breakage of chromosome fragments, the unbalanced exchange of chromatid segments, or damage to the mitotic spindle.

Antiproliferative capacity and genotoxic potential on cell division in *A. cepa* L. of different plant extracts are due to the interaction of various chemical components, present in high concentrations, which cause inhibitory effects on the cell cycle [77,108]. Toxic substances can also affect interphase cells by blocking DNA and protein synthesis in the nucleus [71]. This is evidenced by the presence of micronucleus in interphase and prophase cells. The micronucleus has a similar structure but a reduced size compared to the main nucleus. In the interphase daughter cells, a series of nuclear abnormalities can be identified, such as lobulated nuclei, nuclei with nuclear buds, polynucleated cells or minicells.

**Figure 7:**
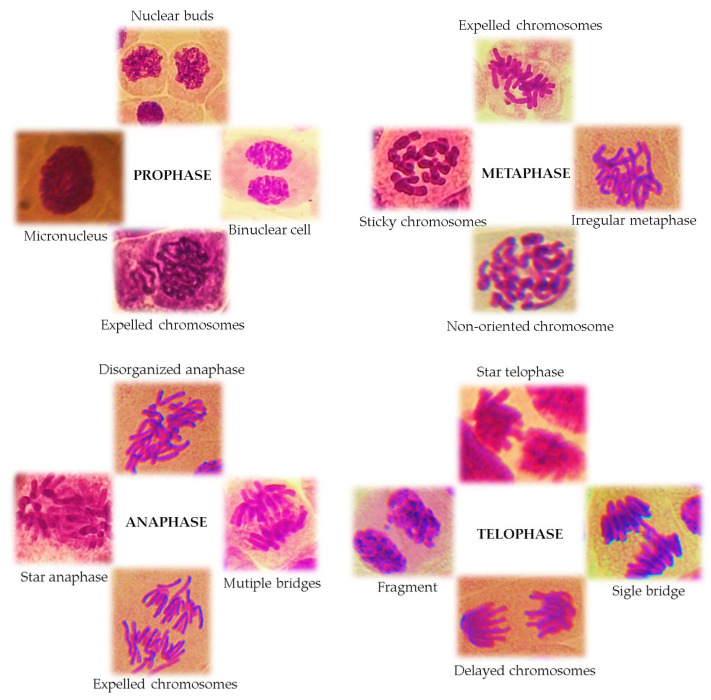
Different chromosomal aberrations in *Allium cepa* L. roots (images from own research).

Some types of chromosomal aberrations in *Allium cepa* L. roots induced by various cytotoxic and genotoxic agents are presented in Figure 7.

Within the *Allium cepa* test, in addition to microscopic parameters, macroscopic parameters can be also measured [30]. The most important macroscopic parameter is the root growth length RGL (the average root length for each sample) [67]. Thus, the percentage of root growth inhibition in the tested extracts compared to the control can be calculated [98,101]. Additionally, the EC50 (effective concentration at which root growth is 50% of the control) can be calculated. Inhibition of root elongation greater than 20% was considered evidence of toxicity, based on standard phytotoxicity tests [54]. Other macroscopic root growth parameters, e.g., restriction of leaf growth, can also be evaluated to estimate the toxicity index (turgescence, consistency, color change, root tip shape, presence of swellings, hooks, twists, or necroses) [30,57,98,102,111].

After centralizing the data, the results can be analyzed using various statistical methods such as chi-square (χ^2^) [84,112,113,114] or one-way analysis of variance (ANOVA) [61,91] followed by Tukey’s test [16,115,116], Bonferroni test [87], etc.

## 4. Results of the Cytotoxic and Genotoxic Evaluation of Plant Extracts Using the *Allium cepa* Model: Literature Review

Early research from 1999 was found in the Web of Science database on the assessment of the cytotoxicity of plant extracts. Aqueous extracts of *Allophylus edulis* leaves obtained by decoction, a plant widely used in folk medicine in Argentina, were studied in terms of cytotoxicity and genotoxicity [102]. Yajía et al. reported an important decrease in the MI compared to the control, indicating a significant statistical correlation between the mitotic index (MI) and the root growth length (RGL) and chromosome aberrations (CAs).

After the 2000s, the number of researchers who started to use the *Allium cepa* model as a tool in the toxicological evaluation of plant extracts has continuously increased.

Table 1 presents the cytotoxic and genotoxic studies using *Allium cepa* assay on various plants.

## 5. Discussion

Most studies highlight that a gradual decrease in MI is significantly correlated with an increasing concentration of the tested extracts or with a longer exposure time, indicating the interference of plant extracts in the progression of the cell division cycle. Moreover, an increase in CA compared with the negative control sample may be detected in the highest concentrations.

The presence of cells with different chromosomal aberrations proves the clastogenic or aneugenic effects of some tested plant extracts [16,66,109,120,142,149]. As previously mentioned, clastogenic aberrations cause changes in the structure of chromosomes, which can be observed at all stages of mitosis, especially in the anaphase, telophase and metaphase of cell division. Plant extracts can induce physiological aberrations (stickiness, c-mitosis and stray chromosomes), which are more frequent than clastogenic aberrations (breaks and bridges) [66]. Thus, in numerous studies, the presence of cells with sticky chromosomes [65,66,67,68], anaphase–telophase with bridges [25,64,76,109,118], laggard chromosomes [117], disordered (irregular) anaphase–telophase chromosome fragments [24,66,69,76], vagrant chromosomes [98,117], c-mitosis [98,108,128], binuclear cell [109,127,135], and enucleated (ghost) cells [120] was frequently detected.

Some extracts can also induce aneugenic changes (adhesion, subsequent segregation, multipolarity, chromosome loss) in *A. cepa* L. roots, causing variations in the number of chromosomes in the meristematic cells. The distinction between a clastogenic effect (damage to the structure of chromosomes) and an aneugenic one (a change in the number of chromosomes) can be made by analyzing the sizes of micronuclei that appear in prophase or interphase cells. If the micronuclei are large, this can be considered an aneugenic effect, since they are formed from entire chromosomes, and, if they are reduced in size, they only contain chromosome fragments [109].

If both the MI and the CA percentages are significantly different in relation to the control at the concentrations tested, a usage warning can be made, revealing possible harmful effects on human health [24,88,118,119,128]. If mild cytotoxicity with minor chromosomal aberrations is revealed, it demonstrates that the extracts in question are safe for consumption [77,104,124,129].

The cytotoxic and genotoxic effects of tested extracts can be related to the phytochemicals present in the species and to their synergetic effect [149,152]. The concentration of metabolites in the extracts can be influenced by the extraction method [118]. Thus, phytochemical screening and the quantification of bioactive compounds present in the tested crude extracts performed using various spectrophotometric methods are important [24,90,119,124]. Simultaneous tests of entire extracts as such, as well as of certain compounds from the extracts (e.g., citral and limonene from *Citrus aurantiifolia* essential oil), were reported by Fagodia et al. [122]. Often, in studies involving essential oils, the observed effects are explained on the basis of their main compounds. Pawlowski et al. [149] state that, even though the major compound of the essential oils from *Schinus molle* and *Schinus terebinthifolius* is α-pinene, the results observed on cell division in onion meristematic cells may be due to both major and minor compounds, rather than a single compound, probably acting synergistically. The presence of monoterpenes or the combined synergistic effect of different monoterpenes in essential oils on the mitotic index of *A. cepa* L. has also been reported in other studies [85,152], without mentioning the specific action of a particular compound. In the study of Cavalcante et al. [87], it was not the entire extract that was tested on onion roots, but rather 2-oleyl-1,3-dipalmitoyl-glycerol, a separate compound from *Platonia insignis* extract.

The findings of the studies showed that the level of cytogenotoxicity of plant extracts on onion meristematic cells can be influenced by several factors related to both the extraction process for the tested samples and the experimental conditions of the *Allium* test itself.

Parameters such as the plant material conditioning (state), solvent, temperature, time, and extraction method influence the content of extracts in active principles, which may lead to different results in terms of cytotoxicity and genotoxicity.

In the extraction process, the plant material (aerial part, leaf, stem, inflorescence, rhizome, bark, etc.) can be used both fresh [85,88,94,112,119,145] and dried, crushed [137] or in powder form [66,82,120]. Drying is carried out using classical methods (oven) or modern methods (freeze drying) [116].

Concerning the solvent, water is the most frequently employed solvent, resulting in aqueous extracts either by infusion, decoction or maceration. Hot water is often used, but so is cold water [59,72]. The aqueous extracts (infusion, most often) are prepared as they would be made at home by the general population [24,64,68,98]. Studies are also reported on hydroalcoholic [82,84,118,131], methanolic [67,69,76,89,124], hexanic [87] or dichloromethane [61] extracts. The essential oils obtained from various plants are also subjected to toxicological research using the *Allium* test [79,85,91,94,95,115,116,119,122]. The cytotoxicity and genotoxicity effect of different concentrations of latex from *Hancornia speciose* or *Jatropha curcas* L. are reported [129,133].

Furthermore, in several studies, comparative cytotoxic and genotoxic evaluations are performed between aqueous extracts and alcoholic extracts [23,118,123,134,137] or between aqueous extracts and essential oils [70,81,94] from the same plant source.

The experimental conditions of the *Allium* test can vary. Usually, different concentrations/dilutions of the studied extracts are tested, and exposure can occur at different times.

Some studies reported that the onion roots were immersed directly in plant extracts without any dilution [24,64,68,98] or the tested concentrations were established according to doses that are recommended in alternative medicinal usage by the general public [141].

In parallel with the *Allium cepa* assay, several studies analyze cytotoxicity using tests on animal cells (e.g., bone marrow of rat cells) [104,109,136], human cells (e.g., lymphocytes) [91], tumor cell lines [138], and brine shrimp (*Artemia salina*) [87,132,138]. There are some studies that report tests on different weeds [79,85,122] to establish the phytotoxicity of the tested plant extracts. In addition to the *A. cepa* assay, recent research on *Platonia insignis* extracts has reported tests on different insects or aquatic crustaceans’ species [87]. Most often, the results concerning the toxicology of plant extracts using different kinds of tests are similar to those obtained with the *Allium* test.

Additionally, the examination of antigenotoxicity can establish the potential protective effect of plant extracts against alterations or mutations induced in the genetic material by various compounds. The antigenotoxic effect of some herbal extracts has been tested in relation to the genotoxicity induced by mutagenic substances such as hydrogen peroxide [120,141], methyl methanesulfonate [109,125] or lead nitrate [150] on onion meristematic cells.

## 6. Conclusions

The side effects associated with the consumption of plants are essentially influenced by the dosage and frequency of their use. Since bioactive compounds and their interactions can lead to toxic effects, there is a constant need for scientific knowledge regarding the efficacy and safety of medicinal herbs. It is important to establish, with accuracy, what the ideal and safe concentrations are for the use of these plants.

The present review is intended as proof that the *Allium cepa* model constitutes an important cytogenetic tool in the process of evaluating the biosafety potential of herbal extracts. This plant-based assay allows for the detection of the possible cytotoxic and genotoxic effects induced on onion meristematic cells, as a prompt step in the evaluation process.

**Figure 8:**
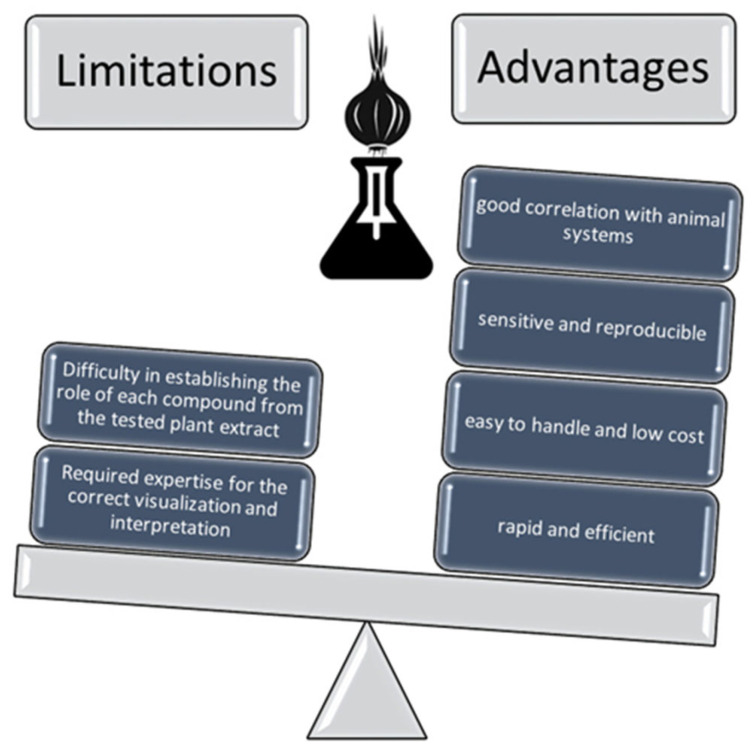
Advantages and limitations of the *Allium cepa* model.

The *Allium cepa* model is not a perfect tool, presenting some limitations (Figure 8). Considering that plants extracts represent a complex mixture of biomolecules, it is difficult to establish the selective influence of each compound on cell division. Some of them may have cytotoxic and/or genotoxic effects, while others may possess cytoprotective and/or antigenotoxic properties. Moreover, the use of the test requires expertise in the correct visualization and interpretation of mitotic phases and chromosomal abnormalities. Based on the research reviewed, it most often emerged that, for a more in-depth study of the cytotoxicity of plant extracts, other tests must be performed, as a complement to the *Allium* test.

Nevertheless, the *Allium cepa* bioassay is a successful test that is used because of its many advantages: simplicity in implementation, cheapness, high sensitivity, and reproducibility. Moreover, the results provided by this biomarker are in good correlation with other accessible systems like animals, cell lines, etc. The ecological significance of using this model, which can be considered environmentally friendly, must also be highlighted. The advantages of the *Allium* test are far greater than the disadvantages presented.

It is concluded that the *Allium cepa* test is a very convenient tool, especially in the preliminary screening of the cytotoxic and genotoxic effects of various plants frequently used in traditional medicine, from which aspects related to their biosafety potential can be established.

## 7. Future Directions

Although the *Allium cepa* test cannot completely replace other cytotoxicity tests, combining it with the analysis, identification and quantitative determination of bioactive compounds in the analyzed plant extracts could lead to clearer information about the causal compounds of the observed effects.

The application of the *Allium cepa* model, however, indicates significant scientific discoveries, and new adaptations of the test, as well as its standardization, could lead to countless possibilities for its use, avoiding other types of more laborious tests, including those on animals.

In recent years, cytotoxicity research has tended to use and develop sustainable testing systems such as the *Allium cepa* assay, with the aim of completely or partially replacing a large percentage of sophisticated experiments involving high costs and environmentally unfriendly methods.

## Figures and Tables

**Table 1:** Application of the *Allium cepa* model for the evaluation of cytogenotoxic effects of plant extracts: literature review (in alphabetical order of plant material species).

Plant Species	Type of Extract/ Plant Organ	Findings	Ref.
*Achyrocline* *satureioides*	aqueous extracts/ dried aerial parts	Absence of genotoxicity was revealed High concentrations induced reversible cytotoxicity effect No associated mutagenicity was demonstrated	[72]
*Adhatoda vasica* *Carica papaya* *Clinacanthus nutans*	aqueous and methanolic extracts/ dried powdered leaves	Cytotoxic effect of methanolic extract of *A. vasica* (at low concentration) No cytotoxic potential in the case of *C. papaya* and *C. nutans* species, regardless of the type and concentration of the extracts tested Moderate mutagenic potential of methanolic extract of *C. papaya* and aqueous extract of *C. nutans*, both in high concentrations	[117]
*Allophylus edulis*	aqueous extracts (decoction)/ fresh leaves and young stems	A dose-dependent effect on MI and CA Between MI and the RGL and CA, there exists a significant statistical correlation	[102]
*Aloysia gratissima*	aqueous extracts (infusion) and essential oils/fresh leaves	Only MI was calculated Antiproliferative effects of tested extracts were observed	[94]
*Aloysia gratissima* (Gillies & Hook.) Tronc.	aqueous and hydroethanolic extracts/leaves	The greatest genotoxic effect presented by the hydroalcoholic extract would be due to its higher concentration of metabolites Controlled consummation is suggested	[118]
*Alpinia zerumbet* (Zingiberaceae)	essential oils/ fresh leaves	MI and CA were significantly different compared to the control Potential damaging effects on human health	[119]
*Amaranthus* *spinosus*	aqueous extracts/ dried powdered leaves	MI decreases with increasing concentration and duration of exposure Various clastogenic and non-clastogenic aberrations were observed	[120]
*Ambrosia* *artemisiifolia* L.	essential oils/ dried aerial part	MI decreased considerably for all tested concentrations, but without significant differences compared to the positive control Different types of CAs were induced The results of simultaneous tests on different weeds suggest that the essential oils have bioherbicide potential	[79]
*Aristolochia indica*	aqueous extracts/ dried powdered roots	Significant inhibition of mitosis in onion root tips Chromosome breaks induced by all analyzed samples Even though the extracts possess pharmacological properties as nephrotoxic agents, it can cause adverse toxicological effects at cellular as well as genetic levels	[73]
*Artemisia* *verlotorum*	aqueous extracts (infusion)/ dried leaves	Genotoxic and antiproliferative effects were indicated Only after selecting the appropriate concentrations, the infusions can be suitable for medicinal purpose	[77]
*Asplenium* *scolopendrium* L.	ethanolic extracts (prior to and after silver nanoparticles phytosynthesis)/fresh leaves and rhizome	The extracts from leaves exhibit a mito-stimulating effect augmented by the synthesis of silver nanoparticles The mitotic inhibitory effects of rhizome extracts supplemented with silver nanoparticles are highlighted An increase in the variability and frequency of chromosome aberrations related to the phytosynthesis of silver nanoparticles The exposure period is important in interpreting the biological reactivity	[75]
*Averrhoa carambola* L. *Cissus sicyoides* L. *Syzygium cumini* (L.) Skeels	aqueous extracts (infusion)/ fresh leaves	The infusion was prepared as it is usually made at home Simultaneous tests on the bone marrow cells of Wistar rats Results showed that the studied teas did not alter the cell cycle Corroboration with the results obtained in the animal test system	[104]
*Azadirachta indica* (A. Juss) *Carica papaya* (Linn.) *Cymbopogon citratus* (DC Stapf.) *Mangifera indica* (Linn.) *Morinda lucida* (Benth.)	aqueous extracts (decoctions or squeezed extracts)/leaves or bark	MI is concentration dependent At 20% concentration of *M. lucida* and *C. papaya* extracts, no dividing cells were observed Mitotic spindle disturbances were reported for all tested extracts The studied extracts may have mitodepressive effect on the cell division of *A. cepa* L.	[64]
*Bacopa monnieri*	aqueous extracts/dried powdered plants	The chemotype with highest bacoside A content exhibited no significant inhibition on MI The absence of genotoxicity of the plant extracts associated with acetylcholinesterase-inhibitory activity indicated its safe use	[74]
*Capparis spinose* L.	aqueous extracts (decoction)/flower buds	Growth retardation was observed Significant decrease in MI was detected in root-tip cells treated with aqueous extract before and after the ethyl methane sulfonate exposure Non-genotoxic effect and anti-mutagenic potential against the damage caused by ethyl methane sulfonate at chromosomal level	[121]
*Centella asiatica* Linn.	aqueous extracts (decoction)/ground dried leaves	Tests on diluted samples obtained from the stock extract by decoction Not cytotoxic effect for the diluted samples except at 100% concentration	[71]
*Chamomilla recutita* (Asteraceae)	aqueous extracts (infusions) and essential oil/inflorescences	The plant was cultivated with homeopathy Infusions and EOs of chamomile cultivated with homeopathy do not induce a genotoxic effect on onion roots	[81]
*Citrus aurantiifolia*	essential oils/ fresh leaves	Alterations in MI and mitotic phases were observed Several CA were induced by the tested samples Simultaneously, phytotoxicity tests on some selected weed species were assessed Cytotoxicities of limonene and citral (two components of the studied EOs) were separately evaluated and citral showed the highest toxicity	[122]
*Clerodendrum inerme* L. Gaertn.	aqueous extracts/ leaves powder	The colchicine was used for comparative metaphase-arresting activity analysis Similar colchicine mitotic depression was induced by the studied extracts	[114]
*Clerodendrum inerme* (L.) Gaertn *C. viscosum* Vent.	aqueous and methanolic extracts/fresh leaves	Blockage of onion cells into mitosis and induction of some chromosomal aberrations The manifestation of effects was differential in relation to extract types and concentrations Methanolic extracts may be more useful for therapeutic studies due to a lack of chromosomal aberrations in most of the administered doses	[123]
*Costus spiralis* (Jacq.) Roscoe	aqueous extracts/ dried and powdered leaves and stems	The extracts presented cytotoxicity, but no mutagenicity was observed The leaves’ aqueous extract presented anti-genotoxicity, reducing the sodium azide cytogenotoxic effects The stems’ aqueous extract enhanced the sodium azide cytogenotoxicity in some conditions Empirical utilization of aqueous extracts should be avoided	[111]
*Crinum asiaticum*	aqueous extracts/ pulverized leaves powder	Concentration-dependent decrease in MI Various cytological aberrations were induced The extracts may contain phytochemicals with cytostatic and cytotoxic effects Indiscriminate use of infusion should be restricted	[101]
*Curcuma amada* *Curcuma karnatakensis*	methanolic extracts/dried leaves	Mitoinhibition and leaf extract interference in cell division cycle progression Negligible CA induced by both the extracts, indicating the lower genotoxic potential of leaf extracts Can be used to prepare herbal medicines and safe for consumption	[124]
*Dionaea muscipula* Ellis	dichloromethanolic extracts/fresh and dried leaves cultivated in vitro	Inhibition of *A. cepa* and *L. sativa* seed germination and growth cytotoxic and genotoxic effects in the *A. cepa* L. roots Possibility of developing bioherbicides based on the extracts	[61]
*Distephanus* *angulifolius*	aqueous extracts/ dried leaves powdered	There was a dose-dependent decrease in the MI when increasing the concentration of the extract CAs increased with an increase in the concentration of the test solution The types of CAs induced by the extracts suggest that it could be clastogenic	[66]
*Erythrina velutina*	aqueous extracts/dried and powdered leaves	Anti-genotoxicity of tested extracts in pre-treatment conditions (first exposure to aqueous extracts and then to methylmethanesulfonate) Protective effects were observed in simultaneous conditions (onion roots exposed to both aqueous extracts and methylmethanesulfonate) The results demonstrate the chemopreventive potential of tested extracts	[125]
*Euphorbia hirta*	methanolic extracts/dried plant material	A dose-dependent increase in chromosome aberrations Significant genotoxic and mitodepressive effects at high concentrations	[69]
*Ficus benghalensis* L.	crude methanol extract and subsequent fractions/dried stem bark material	A decrease in MI in a dose-dependent manner was observed n-butanol fraction showed the strongest antimitotic activity, being the appropriate extract for isolation of bioactive anticancer compounds	[126]
*Garcinia cambogia* (Gaertn.) Desr.	extracts in different solvents (water, ammonia, hexane, chloroform)/ fresh male flower powder	In all samples tested, the suppression of mitotic activity in *A. cepa* L. cells was observed The MI decreased with increasing solvent concentrations in the extract The percentage of cells with chromosomal aberrations decreases with increasing solvent concentrations in the extracts	[127]
*Grewia lasiocarpa*	aqueous extracts/ leaves and stem bark	Dose-dependent root growth inhibitory activity The tested extracts exhibit relatively low cytotoxic effects Administration should be undertaken with caution, as the presence of CA was reported	[128]
*Hancornia speciosa* Gomes	latex	No cytotoxicity was revealed under the testing conditions Low incidence of CA indicated no genotoxic effects Possesses the potential for use in medicine as it is not harmful to human health	[129]
*Heinsia crinata*, *Justicia insularis Lasianthera africana*	ethanolic extracts (maceration)/dried powdered leaves	Three different concentrations were tested Methotrexate was used as the positive control All tested extracts possessed cytotoxic effects and induced CAs and micronuclei	[82]
*Helichrysum* *italicum* (Roth) G. Don *Lavandula angustifolia* Mill. (OmniaNatura, B&H)	essential oils (commercial products)	The frequency of CAs was increased in comparison with controls Higher number of apoptotic cells Cyto/genotoxic effects in both plant and human cells (lymphocytes), as well as antimicrobial properties	[91]
*Hymenaea* *stigonocarpa*	hydroalcoholic extract/dried and powdered bark	Antiproliferative effect when compared to the control sample Considerable reduction in CA compared to positive controls Antimutagenic action on onion cells	[84]
*Hymenaea stigonocarpa* Mart. (Fabaceae)	crude aqueous extracts /rhytidome	Even for the lowest concentration (considered suitable for use), a significant antiproliferative action on the cell cycle was found High number of cells in prophase and cytotoxic effects under the studied conditions	[130]
*Hymenaea stigonocarpa* Mart. ex Hayne (Fabaceae, Caesalpinioideae)	hydroalcoholic extracts (macerate)/dried and grounded bark	Ability to inhibit cell proliferation (at a long exposure time) Number of cell abnormalities was not significant To accurately determine its antiproliferative potential, more tests using other systems are needed	[131]
*Hypericum perforatum* L. (St. John’s wort)	aqueous extracts/ dried and grounded leaves	No potential cytotoxic or mutagenic on the onion root meristematic cells Similar, no cytotoxic or mutagenic effect on bone marrow cells from Wistar rats Does not appear to be harmful to the cells of organisms in the studied experimental conditions	[103]
*Hyptis suaveolens*	essential oil/ fresh leaves	A decrease in MI and an increase in CA were observed for the treatment with the highest concentration of EOs Potential natural herbicide (phytotoxic activity of EOs against *Echinochloa crus-galli* weeds)	[85]
*Icacina trichantha* Oliv.	aqueous extracts/ dried leaves	The root growth was significantly inhibited by the extracts compared to the control An increase in cytological effects related to the extract concentration is observed Inhibitory and mitodepressive effects on the cell division	[98]
*Ilex paraguariensis* St. Hil. (*Aquifoliaceae*)	aqueous extracts (infusions and concoctions)/dried leaves and young stems	Both extracts significantly decreased RGL A significant reduction in MI was observed at all concentrations These effects were greater for the commercial products compared to “laboratory” products Results of the *Artemia salina* microwell test indicate that neither of the extracts exhibited cytotoxicity	[132]
*Inula viscosa*	aqueous extracts/leaf	MI was significantly decreased in comparison to the negative control Strong cytotoxic and genotoxic effects induced by the leaf extracts	[65]
*Jatropha curcas* L.	latex	All tested concentrations significantly inhibited the MI of onion meristematic root cells No genotoxic effect was detected at 1% and 0.5% concentrations when compared with the negative control Highly genotoxic activity at 0.1% latex concentrations More studies needed to establish the appropriate quantity for safe and effective use by the population	[133]
*Lagenandra toxicaria* Dalz.	methanolic extracts/ dried rhizomes	The extract induces genotoxicity at higher doses at higher time intervals The results are confirmed by the decreased MI, DNA damage, and lipid peroxidation	[89]
*Lantana fucata* Lindl.	aqueous and hydroalcoholic extracts/dried and grounded leaves	Cytotoxicity of all extracts with a decrease in MI as concentrations increased Hydroalcoholic extracts were the most cytotoxic (due to their abundance in flavonoids and terpenoids) The ingestion of this species in a moderate way is recommended	[134]
*Limonium* *globuliferum*	aqueous extracts/dried powdered leaves, stems and roots	Cytotoxic and genotoxic effects on *A. cepa* L. cells were induced only at high concentrations of the tested extracts Root extracts induced the lowest MI Low concentrations of plant extracts do not affect cell division in onion meristems	[108]
*Luehea divaricata*	aqueous extracts (teas and decoctions)/leaves and bark	No significant genotoxic effect in comparison to the negative control Few chromosomal abnormalities, not differing significantly from the negative control Possibilities of its use in the preparation of antitumor medications	[78]
*Maesa macrophylla* (Wall.) A. DC.	aqueous extract/ fresh ground leaves	The cytotoxic effect depends on the concentration of the extracts and the time of their action in onion roots The lowest MI value was registered at the highest concentration of the extract A high percentage of CA was identified at all tested concentrations	[135]
*Maytenus ilicifolia *(Mart.) *Bauhinia candicans* (Benth)	aqueous extracts (infusions)/ fresh leaves	The infusions did not show a statistically significant mitotic depressive effect on root-tip cells Comparative cytotoxic studies on rat bone-marrow cells were similar to those on meristematic root cells	[136]
*Mimosa pigra* (Fabaceae)	aqueous and ethanolic extracts/dried and crushed leaves and stems	Several variants of extracts were tested Cytotoxic effect of tested samples due to a significant inhibition of MI and an increase in CAs	[137]
*Ocimum basilicum*	aqueous extracts and essential oil/leaves and inflorescences	The influence of the presence or absence of salt stress induction was monitored Cytogenotoxicity of basil extracts is not affected by the induction of salt stress The essential oil has no cytotoxic potential but exhibits genotoxicity	[70]
*Origanum vulgare* L.	aqueous extracts (infusion/decoction) and hydroethanolic extracts/dried leaves	Extraction conditions affected all parameters of the cytogenetic assay In particular, hydroalcoholic extracts as well as extracts obtained via decoction presented cytotoxic and genotoxic effects on onion cells Oregano extracts must be consumed with caution in traditional medicine	[24]
*Origanum vulgare* Lamiaceae. Ssp. *Hirtum*	essential oil/ dried leaves	EOs modifies the onset of mitosis (an increase in the cells in prophase compared to other mitotic phases) No significant change in the percentage of CA in comparison with the negative control Simultaneous anti-inflammatory properties in a mouse-airway inflammation model and the in vitro antimicrobial activities were demonstrated	[95]
*Origanum vulgare* ssp. *vulgare* L.	aqueous extracts (infusions in cold/hot water)	A change in the mitotic phase distribution was observed with an increase in the anaphase index and simultaneous decrease in the telophase index Significant inhibition of mitotic activity indicated the occurrence of a cytotoxic effect Spindle dysfunction and a high percentage of micronuclei in interphase	[59,60]
*Pandanus Odoratissimus f.ferreus* (Y. Kimura) Hatus	aqueous and methanolic extracts/dried and powdered roots and leaves	Significant reduction in the MI of onion root tips with increases in concentration Aqueous extract possesses cytotoxic, antimitotic activities Simultaneous cytotoxicity screening via brine shrimp assay and tumor cell lines revealed its potential use in cancer therapy	[138]
*Parquetina nigrescens*	aqueous crude extracts/fresh leaves	Cytogenotoxicity was observed at high concentrations Consumption with caution is recommended	[88]
*Peganum harmala* L.	methanolic extracts/powdered leaves	For all concentrations tested, a decrease in MI and PI was observed compared to untreated cells The extracts obstructed mitosis in prophase and produced the growth of abnormalities Antimitotic and genotoxic effects on meristematic cells	[76]
*Pfaffia glomerata* Spreng. Pedersen, *Amaranthaceae*	methanolic extract/ small pieces of dried roots	Alterations in the cell cycle and aneugenic and clastogenic effects were observed Similar cytogenotoxic activity of tested extract in animal cells (rodent bone marrow) The consumption of the extract for therapeutic purposes should be done prudently	[109]
*Phyllanthus niruri* L. (Euphorbiaceae)	aqueous extracts (infusions)/ dried leaves	Four concentrations (including the usual one) were used and two exposure times Inhibition of cell division was observed for all concentrations tested and decreased with increasing exposure time Increased CAs compared to the control	[139]
*Phyllanthus tenellus* Roxb	aqueous extracts (infusions)/ aerial part	Cytotoxic effect observed for all extracts obtained from plants grown under different light conditions Genotoxic effect induced only by samples grown in shaded conditions	[80]
*Picea abies* *Pinus nigra*	aqueous extracts and aqueous extracts derived silver nanoparticles/bark	The MI of bark-extract-derived silver nanoparticles was almost half that of the bark extracts used for their synthesis Significant accumulation of cells in prophase Simultaneous test on Gram-positive and Gram-negative bacteria and fungi proved strong antibacterial activities	[140]
*Pimenta dioica*	essential oils/freeze-dried powdered leaves	No sign of genotoxic activity of EOs on meristematic roots of onion Safety towards germinating grains and non-targeted animal species was reported EOs could be used as an environmentally safe larvicidal and biopesticidal compounds	[116]
*Plantago lanceolata*	aqueous extracts/leaves	Lower MI values of aqueous-extract-treated groups after hydrogen peroxide exposure Anti-mitotic and anti-genotoxic effects The protective effect against the oxidative damage induced by hydrogen peroxide may be due to the antioxidant properties of flavonoids	[141]
*Platonia insignis*	hexanic extract (dichloromethane fraction)/seeds	Not the entire extract was tested, but only 2-oleyl-1,3-dipalmitoyl-glycerol (a compound isolated from the dichloromethane fraction of the hexanic extract) The compound demonstrated non-cytotoxic effects at low concentrations At higher concentrations, a slight cytotoxicity in comparison to the negative control was shown Simultaneous tests: no toxicity to *Artemia salina* and no larvicidal activity against *A. aegypti* larvae at the analyzed concentrations and no toxicity	[87]
*Pluchea sagittalis*	aqueous extracts (infusions)/ fresh leaves cultivated in different conditions (in vitro, acclimatized growth chamber, and field)	The antiproliferative capacity was strongly dependent on the origin of the leaves Reduction in MI in samples cultivated in field and in the growth chamber Higher antiproliferative activity in the case of infusion of fresh leaves from field cultivation No decrease in MI for the samples cultivated in vitro, likely due to the lower concentration or type of bioactive compounds accumulated	[86]
*Pogostemon heyneanus* Benth. (Java Patchouli)	aqueous extract (decoction)/dried powder leaves	Cytotoxic effect at all tested concentrations with the increase in extract concentrations and treatment durations Various clastogenic and non-clastogenic aberrations were detected The samples may be effective cytotoxic agents, but supplementary studies are required to characterize and isolate the bioactive agents responsible for this activity	[142]
*Poincianella bracteosa* (Tul.) L.P. Queiroz	aqueous extracts/dried and powdered leaves	The phytochemicals from extracts did not induce aneugenic and clastogenic actions in onion root or blood cells of mice Absence of cytotoxicity and mutagenicity Possible therapeutic applications	[143]
*Polystichum setiferum* (Forssk.) Moore ex Woyn.	methanol and ethanol extracts/frozen leaves and rhizomes	A significant time-related increase in the MI was reported Non-mutagenic effect detected, with no structural and numerical incidence on chromosomal aberrations Potential as antimitotic drugs due to the non-cytotoxic and non-clastogenic effects	[16]
*Psidium guajava* L. *Achillea millefolium* L.	aqueous extracts (infusions)/ fresh leaves	Significant inhibition of cellular division in the onion root-tip cells by the *P. guajava* L. infusion at higher concentrations No noteworthy decrease in MI in onion root-tip cells treated with *A. millefolium* L. Compared to untreated samples, no significant CA after exposure to infusions Similar results in rat cells or in cultured human lymphocytes Cytostatic rather than cytotoxic antiproliferative effect Advice for respecting the recipe for the preparation of infusions and avoiding prolonged use	[144]
*Psychotria myriantha* Mull. Arg. and *Psychotria leiocarpa* Cham. et Schlecht	aqueous extracts (infusions)/ fresh leaves	The onion bulbs were exposed to two different concentrations A decrease in MI with an increase in infusion concentration was observed Both species possess antiproliferative effects on the onion cell cycle *P. myriantha* showed genotoxic activity	[112]
*Pterocaulon* *polystachyum* DC. (Asteraceae)	aqueous extracts (infusions)/ fresh young leaves	A highly significant decrease in the MI of root-tip cells treated with infusions prepared from different populations as compared to those exposed to water The cytotoxic and antiproliferative activity indicates the therapeutic potential of the studied extracts No mutagenic effects on onion root-tip cells	[145]
*Pterodon emarginatus* vogel	crude alcoholic extract standardized in geranylgeraniol/crushed fruits	Increased cell division in onion radicles influenced by the time of exposure Antiproliferative effects of tested extracts Useful as a cytotoxic treatment against infectious agents	[146]
*Pyrostegia venusta*	ethanolic extracts/ dried flowers	The extract reduced chromosomal disorders in onion roots induced by methyl methanesulfonate In a similar way, the extract protected cells from cyclophosphamide (a mutagenic compound), using micronucleus tests in mouse bone marrow Antigenotoxic and antioxidant potential, possibly due to the different flavonoid compounds present in its extract	[147]
*Rosmarinus officinalis* L.	aqueous extracts/ leaves powder	The crude hydroalcoholic extracts possesses chemopreventive activities on mutagenicity induced by methylmethane sulfonate in meristematic cells of onion seeds The most effective were the samples with post and simultaneous exposure	[62]
*Rosmarinus officinalis* L. (Labiatae)	aqueous extracts (decoction followed by infusion)/dried leaves	For all tested concentrations at all exposure times, the MI was significantly reduced A high number of cells in prophase was reported Antiproliferative effect on the cell cycle including at the lowest concentration, which is considered ideal for use	[148]
*Schinus lentiscifolius* March.	essential oils/ air-dried leaves	The essential oil caused a decline in the MI of onion meristematic cells No significant change was noticed in the metaphasic index of onion A variety of genotoxic effects in onion through the occurrence of CA was reported	[115]
*Schinus molle* *Schinus terebinthifolius* (Anacardiaceae)	essential oils/ dried leaves	A significant reduction in MI was observed in the case of *S. terebinthifolius* (82.03%), while, for *S. molle*, it was only 21.05% Similar results were obtained for *L. sativa* Even if α-pinene is the major compound of the EOs, the inhibitory effects observed on onion and lettuce are due to synergetic actions of both major and minor compounds	[149]
*Solenostemon* *monostachyus* (P. Beauv.) Brig. (Lamiaceae)	ethanolic extracts (maceration)/ powdered dried leaves	Three different concentrations were tested and methotrexate was used as positive control Cytotoxic potentials of all tested extracts were revealed Mitodepressive and aneugenic effects in onion and lettuce root meristems	[83]
*Solidago microglossa* DC. (Asteraceae)	aqueous extracts (infusions)/leaves	At the highest concentration, a significant reduction in the MI compared with the control in the case of infusions obtained from three different populations An increase in MI for populations 1 and 3 at concentrations commonly used by people Antiproliferative effect at the highest concentration	[113]
*Spondias mombin* L., *Nymphea lotus* L. *Luffa cylindrica* L.	aqueous extracts (decoction)/leaves and whole plant	Inhibition of root growth by the extracts depending on concentration Mitodepressive effects on cell division for all tested extracts Reduction in CA induced by lead nitrate, so the anti-genotoxic effect is highlighted	[150]
*Tapinanthus* *bangwensis* *Moringa oleifera*	methanol extracts/ ethylacetate fractions/ acetone fractions/ powdered leaves	As the concentration increases, there is a decrease in the MI Acetone fractions are the most cytotoxic, followed by methanol extracts and ethyl acetate fractions Significant increase in sticky chromosomes, followed by bridged and vagrant chromosomes	[67]
*Thottea siliquosa* (Lam.) Ding Hou	methanolic extract/dried and powdered leaves	No significant cytotoxic effect in onion root cells It was found that the extract protects against the toxic effect of ethyl methyl sulfonate The extracts are safe for use and possess genoprotective effects	[151]
*Vernonanthura* *polyanthes*	aqueous extracts (infusion)/dried leaves	Cytotoxic effect in onion root cells was observed at high concentration of the extract (double that commonly used in popular medicine) No genotoxic activity of extracts was observed in the tested conditions Potential use for human medicine	[90]
*Viscum album* different mistletoe host trees: *Abies alba*, *Acer saccharinum*, *Malus domestica*, *Pinus sylvestris*	aqueous extracts/ fresh plant	Simultaneous tests on *Drosophila milanogaster* and some bacteria and fungi species The highest cytostatic effect on *A. cepa* L. and *D. melanogaster* in the case of extract from silver maple host (*A. saccharinum)*	[99]
*Vitex negundo*	essential oils/ fresh leaves	Significant decrease in MI and an intensification of CA in a concentration-dependent manner Genotoxicity was probed using simultaneous comet assays (an increase in DNA damage) Cytotoxic and genotoxic effects of EOs indicate it as a potential phytotoxic agent against weeds	[152]
*Ziziphus* *mauritiana* (Lam)	extracts in different solvents (waters/ethanol/ ethylacetate/hexane)/powdered leaves	The ethanolique extract showed the most important effect on MI MI and CA decreased with increased concentrations of extracts In all four extracts, various CAs were observed	[68]

MI—mitotic index, PI—phase index; CA—chromosomal aberrations; EOs—essential oils.

## Data Availability

Not applicable.
